# Ketoacidosis in type 1 diabetics: we should return to pediatric guidelines

**DOI:** 10.1186/s13613-020-0639-z

**Published:** 2020-02-11

**Authors:** Sébastien Redant, David De Bels, Jacques Massaut, Jacques Devriendt, Xavier Beretta-Piccoli, Rachid Attou, Patrick M. Honore

**Affiliations:** 1 ICU Department, Centre Hospitalier Universitaire Brugmann-Brugmann University Hospital, 4, Place Arthur Van Gehuchten, 1020 Brussels, Belgium; 2 ICU Department, Hopital Universitaire des Enfants Reine Fabiola, Brussels, Belgium

We read with interest the study of Balmier et al. about initial management of patients with ketoacidosis (KA) according to diabetes type [1]. Authors report a high rate of hypoglycemia in type 1 diabetics compared to type 2, secondary diabetes and newly diagnosed diabetes. Authors also report a significantly high rate of hypokalemia in newly diagnosed diabetes, as being the witness of a more profound potassium depletion in the context of a prolonged osmotic diuresis and cetonuria. Balmer et al. advocate for dose-adjustment according to diabetes type. We would like to emphasize on returning to pediatric guidelines in type 1 adult diabetics with KA.

Pediatricians have a constant concern of closely monitoring the decrease in blood glucose because there is a controversy over the association between the decrease in blood glucose and osmolarity which could be responsible for cerebral edema. The original cerebral edema has been characterized in animal models. During acute hyperglycemia, occurs intracellular dehydration thwarted by the appearance of idiogenic osmoles in the brain different from lactate, urea, sorbitol, amino acids, and myoinositol. During a too rapid correction of the glycemia then follows an osmotic imbalance with an intracellular water entry [2]. In the study of Edge et al., it was shown that there was an association between the insulin dose given during the first 2 h and the risk of brain edema [3]. Several pediatric guidelines have eliminated insulin administration during the first hour, recognizing that fluid administration alone reduces blood glucose by improving renal perfusion and osmolar charge clearance [4]. Boluses of 10–20 ml/kg are administered in order to restore an effective blood volume and then a maintenance infusion equivalent to 1500 ml/m^2^ body surface area [5].

Similarly, pediatric guidelines on ketoacidosis management are against the administration of an initial bolus of insulin [5]. An initial bolus was thought to counteract the relative insulin resistance present in diabetic KA [6]. An adult study has shown that administering an initial bolus does not change the rate of decrease in blood glucose, anion gap variation, and hospital length of stay. More patients developed hypoglycemia in the bolus group than in the control group, but did not achieve statistical significance due to lack of potency [6].

Regarding the insulin dose, Nallasamy et al. compared 0.1 IU/kg versus 0.05 IU/kg in a randomized study. They observed no difference in duration of acidosis, with less hypoglycemia and hypokalemia in the low-dose group [4]. In pediatric patients, the continuous IV insulin dose remains constant (0.1 IU/kg) until glycemia reaches 150 mg/dl and is then reduced by half (0.05 IU/kg). Glucose management is achieved by glucose administration when blood glucose reaches 300 mg/dl [5]. Maintaining insulin constant could have an effect on serum potassium stability which would depend only on acidosis and osmolarity. Reducing insulin doses would allow safer management of insulin leakage, electrolytes and osmolarity.

At the sight of the Balmier study our proposal would be, in type 1 diabetes ketoacidosis, to start insulin after 1 h of hydration without bolus at a constant rate of 0.05 IU/kg and manage glycemia with glucose infusion. Further randomized studies would be needed to verify this proposal in adults.

## Data Availability

Not applicable.

## References

[1] Balmier A, Dib F, Serret-Larmande A, De Montmollin E, Pouyet V, Sztrymf B, Megarbane B, Thiagarajah A, Dreyfuss D, Ricard JD, Roux D (2019). Initial management of diabetic ketoacidosis and prognosis according to diabetes type: a French multicentre observational retrospective study. Ann Intensive Care..

[2] Arieff AI, Kleeman CR (1973). Studies on mechanisms of cerebral edema in diabetic comas. Effects of hyperglycemia and rapid lowering of plasma glucose in normal rabbits. J Clin Invest..

[3] Edge JA, Jakes RW, Roy Y, Hawkins M, Winter D, Ford-Adams ME (2006). The UK case–control study of cerebral oedema complicating diabetic ketoacidosis in children. Diabetologia.

[4] Nallasamy K, Jayashree M, Singhi S, Bansal A (2014). Low-dose vs standard-dose insulin in pediatric diabetic ketoacidosis: a randomized clinical trial. JAMA Pediatr..

[5] Rosenbloom AL (2010). The management of diabetic ketoacidosis in children. Diab Ther..

[6] Goyal N, Miller JB, Sankey SS, Mossallam U (2010). Utility of initial bolus insulin in the treatment of diabetic ketoacidosis. J Emerg Med.

